# CIRSE Standards of Practice for the Classification of Complications: The Modified CIRSE Classification System

**DOI:** 10.1007/s00270-025-04200-w

**Published:** 2025-10-16

**Authors:** D. Filippiadis, P. L. Pereira, K. A. Hausegger, A. G. Ryan, C. A. Binkert

**Affiliations:** 1 Second Department of Radiology, School of Medicine, National and Kapodistrian University of Athens, “Attikon” University General Hospital, Athens, Greece; 2 Center for Radiology, Minimally-Invasive Therapies and Nuclear Medicine, SLK Kliniken GmbH Heilbronn, Heilbronn, Germany; 3 Ruprecht Karls University of Heidelberg, Heidelberg, Germany; 4 Faculty of Eberhards-Karls-University, Tübingen, Germany; 5 Danube Private University, Krems, Austria; 6 Uniklinikum Salzburg Division of Interventional Radiology, Müllner Hauptstraße 48, 5020 Salzburg, Austria; 7 Division of Interventional Radiology, Department of Radiology, University Hospital Waterford, Ardkeen, Waterford City, Ireland; 8 Royal College of Surgeons in Ireland, Dublin, Ireland; 9 University College Cork, Cork, Ireland; 10 Department of Radiology and Nuclear Medicine, Cantonal Hospital Winterthur, Brauerstrasse 15, 8401 Winterthur, Switzerland; 11 Interventional Radiology Clinic at MRI Zürich, Hofwiesenstrasse 349, 8050 Zürich, Switzerland

**Keywords:** Complications, Radiology, Interventional, Classification

## Abstract

The CIRSE classification system was published in 2017 aiming to standardize the reporting of complications by avoiding subjective definitions and an excessive number of grades. The original system described 6 grades: Grade 1: complication could be solved within the same procedure, Grade 2: unplanned prolonged hospitalization < 48 h without an additional therapy, Grade 3: additional therapies needed or hospitalization > 48 h, but no sequelae, Grade 4: mild sequelae beyond hospitalization, Grade 5: severe sequelae requiring assistance in daily life, and Grade 6: death.

Following initial evaluation through a validation process performed during the International Conference on Complications in Interventional Radiology (ICCIR 2023), a need to modify the system was identified, specifically to stratify grades 1 and 3 based on the degree of impact on the patient in terms of success or failure of the procedure (Grade 1) and on the duration of hospital stay (Grade 3). Thus, Grade 1 was subdivided into 1a and 1b: 1b describing situations when the complication was resolved within the same procedure, but the intended procedure was not completed and Grade 3 subdivided into 3a: Hospital stay > 48 h, but < 2 weeks and 3b > 2 weeks to depict the complications that resulted in the most time- and resource consuming hospitalizations. It remained the case that no permanent sequelae are observed in grade 3.

This initial testing of the modified classification system in an international IR complication meeting showed high reliability and strong inter-observer agreement. The modified CIRSE system for classification of complications addresses the shortcomings of the original system while remaining easy to use with clear and objective parameters describing the clinical outcomes after a complication.

## Introduction

Ensuring patient safety, avoiding complications and the assurance of high-quality treatment delivery and outcomes are the major pillars of successful Interventional Radiology (IR) practice. Improved outcomes are closely related to uniform reproducible practice including, among others, report standardization. Health care policies and reimbursement strategies for therapeutic procedures are based on the evaluation of outcome data including, among others, safety, efficacy, and cost-effectiveness. This makes clear and consistent reporting of complications with a reproducible and validated categorization system a prerequisite. To date, complications have been reported with a wide variety of different systems using different definitions and scores [1–7]. Aiming to provide a reproducible, easy-to-use grading of complications, the Cardiovascular and Interventional Radiological Society of Europe (CIRSE) published the first version of the CIRSE classification system in 2017 [1]. This system was designed specifically for reporting complications secondary to image-guided minimally invasive therapies. In addition to procedural adverse events (AE), the sequelae (i.e., any negative outcome due to the procedure) after hospitalization were also recorded. The CIRSE classification system for complications combines outcome, presence of complication, effect on hospitalization, and severity of a specific complication and the sequelae in a patient’s everyday life.

To evaluate the 2017 CIRSE classification system, a validation process was performed during the International Conference on Complications in Interventional Radiology (ICCIR 2023). During this conference, all participants (which included two observers for the survey, faculty, and delegates with varying degrees of experience) ranked a total of 30 presented complications using the 2017 CIRSE classification system. During this validation process, the CIRSE classification system was evaluated for its user-friendliness and ease of understanding. In addition, the impact of different levels of familiarity with the classification system was also analyzed. For this purpose, the two observers were considered highly familiar with the CIRSE system as they had served as members of the initial publication’s author group. On the other hand, the presenters of the ICCIR 2023 meeting were asked to familiarize themselves with the system and grade the complication upfront (medium familiarity) while the audience members were informed and asked to grade for the survey during the meeting (low level of familiarity) [8]. Inter-observer agreement and inter-rater reliability were excellent, while a mean agreement of 68.39 ± 20.59% was found on evaluation of audience voting results. Inconsistencies were observed in audience voting for Grades 1 and 3 [8] and these prompted the sub-division in the modified system.

The aim of this SOP document is to describe the changes made to improve the existing system, and in the process, compare the modified CIRSE classification system with other frequently used grading systems.

## Modifications of the CIRSE Classification

The goals of the modified CIRSE classification system were to standardize reporting of complications retaining the simplicity and reliability of the original system while refining the original version to be more specific and permit the recognition of an uncompleted treatment and the downgrading of a moderately prolonged hospital stay. In terms of timing, complications in the modified CIRSE system are still described in relation to the procedure, i.e., intra-procedural, peri-procedural, and delayed. In agreement with the surgical literature, late complications are defined as those observed more than 30 days after the procedure [9]. The first adaptation was to acknowledge that not completing a planned therapy due to a complication should specifically be graded because it leaves the patient untreated. This situation is described in the new Grade 1b.

The second adaptation is the distinction between *moderately* prolonged and *substantially* prolonged hospitalization after a complication. A hospitalization > 2 weeks is presumed to imply a severe procedural complication, which is not only difficult for the patient but also puts a burden on health care resources [10–12]. A hospitalization between 48 h and 2 weeks, on the other hand, is likely less severe and is potentially contributed to by patient factors such as pre-existing morbidities [10–12]. Thus, the modified system distinguishes between grade 3a: > 48 h but < 2 weeks and 3b: hospitalization > 2 weeks.

These modifications have been applied as a result of the testing of the original CIRSE classification system and feedback received during ICCIR 2023.

## The Modified CIRSE Complication System

See Table 1.

**Table 1:** Modified CIRSE Classification System for Complications Reporting

Grade	Description
1a	Complication during the procedure which could be resolved within the same session and the intended procedure was completed; no additional post-procedural therapy, no post-procedure sequelae, no deviation from the normal post-procedural course
1b	Complication during the procedure which could be resolved within the same session, but the intended procedure was not completed; no additional therapy, no post-procedure sequelae, no deviation from the normal post-procedural course
2	Prolonged observation including overnight stay (as a deviation from the normal post-therapeutic course, < 48 h); no additional post-procedure therapy, no post-procedure sequelae
3a	Additional post-procedure therapy or prolonged hospital stay (> 48 h but < 2 weeks) required; no post-procedure sequelae
3b	Additional post-procedure therapy or prolonged hospital stay (> 2 weeks) required; no post-procedure sequelae
4	Complication causing permanent mild sequelae (resuming work and independent living)
5	Complication causing permanent severe sequelae (requiring ongoing assistance in daily life or additional long-term treatment)
6	Death

## Merits of the New CIRSE System Compared to Others

The modified classification system remains easy to use with high objectivity because of the clear and measurable parameters. It addresses the shortcomings found in grades 1 and 3 of the original system. The modified CIRSE classification system for reporting complications may be easily adopted into clinical practice and is amenable to further development and revision through iterative processes of validation. It uses the term complication instead of either *adverse effect or medical error. Adverse effect* is typically used in the pharmacological context to describe an undesired occurrence resulting from taking a medication in the correct dosage whereas *medical error* is used to describe an undesired occurrence related to unsuitable or incorrect dosage). Furthermore, the term *adverse event* has been defined as “an unintended injury or complication caused by medical management resulting in measurable disability, prolonged hospital stays, or death” [13, 14]. *Complications*, on the other hand, can be defined as any symptomatic or asymptomatic deviation from the intended post-therapeutic course that is not inherent in the procedure and does not comprise a failure to cure; thus, complications represent a subset of adverse events that occur in IR [14, 15].

With respect to reporting complications, the modified CIRSE classification system is easy to apply, avoiding complex definitions and an excess of grades. It is reproducible and flexible, providing broad-based, general criteria that can be applied in a uniform way to the various complications encountered. It is characterized by homogeneity across systems, e.g., avoiding categorization of complications into vascular or non-vascular which could lead to unnecessary complexity and potentially hamper future data comparisons in terms of statistical significance. The focus of the modified CIRSE classification system is on patient outcomes, and the system can be used by all physicians performing image-guided procedures. The system can be used either directly post-intervention or retrospectively when reporting or publishing data. The classification approach utilized in the modified CIRSE classification system emphasizes the combination of outcome, presence of complication, impact on hospitalization, and severity of a specific complication and sequelae in a patient’s everyday life [8].

## Comparison of the Modified CIRSE System in Relation to Other Systems

The Common terminology criteria for adverse events (AE) reporting defines an AE as any unfavorable and/or unintended symptom, or disease temporally associated with the use of a medical treatment or procedure that may or may not be considered related to the medical treatment or procedure; it is a descriptive terminology grading a classification by anatomical or physiological system, etiology, or purpose [5]. Although the system is used quite often, it is not easily applied to IR procedures and therapies. We would like to compare and contrast the modified classification system to others such as the Clavien–Dindo system [4], the adverse event classification of the SIR [2, 3] and the modified Rankin scale (mRS) [6].

The modified Clavien–Dindo classification system is used for reporting surgical outcomes, correlating the severity of a complication with the degree of medical therapy and effort required for its reversal or management [4]. The Clavien–Dindo classification focuses on the type of treatment used to treat the complication rather than the hospitalization time. Grade 1 (both 1a and 1b) in the modified CIRSE classification would likely not be mentioned in the modified Clavien–Dindo system. Grade 2 in the CIRSE system is equivalent to Grades I and II of the Clavien–Dindo system, whereas Grade II (transfusion and parenteral nutrition) would rarely apply to IR patients. Grade 3 in both systems relates to additional treatment necessary to solve the complication. In the CIRSE system, the degree of severity is defined by hospital stay, whereas in the Clavien–Dindo system, the sub-division is based on the additional treatment requiring or not requiring general anesthesia. Grade IV in the Clavien–Dindo system refers to the need for ICU whereas in the CIRSE system, the focus is on the sequelae after hospitalization. Death as the ultimate complication is Grade V in the Clavien–Dindo and 6 in CIRSE.

The SIR adverse event classification system has two parts [2]. Part A describes the adverse event. Part B is meant to help in the analysis of the adverse event by looking at causality, patient risk factors, preventability, and management. This part B is primarily for scientific purposes rather than for use in clinical practice. Part A has 5 severity levels. The first level is equivalent to CIRSE grade 1a. Level 2 and level 3 are similar to the corresponding CIRSE grades but are much less specific in terms of what they are based on, leaving more room for subjectivity. Level 4 is similar to Clavien-Dindo IV, i.e., needing ICU care. The most severe level is death as in all the other classification systems.

The modified Rankin scale (mRS) is not designed to grade complications; rather, it has a strong focus on grading outcomes after a stroke [6]. To that extent, the CIRSE classification is similar. Score 3 of the mRS has moderate disability similar to CIRSE grade 4. Scores 4 and 5 of the mRS describe severe disability, similar to CIRSE grade 5. Again, the highest grade is death.

## Conclusion

The modified CIRSE classification system retains the ease of use and objectivity of the original system, but has improved the specificity of grades 1 and 3 by adding a grade for complications which were resolved within the same procedure but without the planned procedure’s successful completion (1b) and separation by length of hospitalization shorter (3a) or longer than 2 weeks (3b) in order to distinguish the severity of the complication and the resultant healthcare costs.

Ultimately, the adaptation of this modified CIRSE classification system in the clinical practice of minimal invasive procedures will demonstrate its value and utility.

## Abbreviations

AE: Adverse events
CIRSE: Cardiovascular and Interventional Radiological Society of Europe
ICCIR 2023: International Conference on Complications in Interventional Radiology
ICU: Intensive care unit
mRS: Modified Rankin scale
SIR: Society of Interventional Radiology

## References

[1] Filippiadis DK, Binkert C, Pellerin O, Hoffmann RT, Krajina A, Pereira PL. CIRSE quality assurance document and standards for classification of complications: the CIRSE classification system. Cardiovasc Intervent Radiol. 2017;40(8):1141–6. 28584945 10.1007/s00270-017-1703-4

[2] Baerlocher MO, Nikolic B, Sze DY. Adverse event classification: clarification and validation of the Society of Interventional Radiology specialty-specific system. J Vasc Interv Radiol. 2023;34(1):1–3. 36244632 10.1016/j.jvir.2022.10.011

[3] Khalilzadeh O, Baerlocher MO, Shyn PB, Connolly BL, Devane AM, Morris CS, et al. Proposal of a new adverse event classification by the Society of Interventional Radiology Standards of Practice Committee. J Vasc Interv Radiol. 2017;28(10):1432–7. 28757285 10.1016/j.jvir.2017.06.019

[4] Clavien PA, Barkun J, de Oliveira ML, Vauthey JN, Dindo D, Schulick RD, et al. The clavien-dindo classification of surgical complications: five-year experience. Ann Surg. 2009;250(2):187–96. 19638912 10.1097/SLA.0b013e3181b13ca2

[5] US Department of Health and Human Services, National Institutes of Health, National Cancer Institute Common Terminology Criteria for Adverse Events (CTCAE) v5: https://ctep.cancer.gov/

[6] Quinn TJ, Dawson J, Walters MR, Lees KR. Exploring the reliability of the modified rankin scale. Stroke. 2009;40(3):762–6. 19131664 10.1161/STROKEAHA.108.522516

[7] Dindo D, Demartines N, Clavien PA. Classification of surgical complications: a new proposal with evaluation in a cohort of 6336 patients and results of a survey. Ann Surg. 2004;240(2):205–13. 15273542 10.1097/01.sla.0000133083.54934.ae PMC1360123

[8] Filippiadis DK, Pereira PL, Hausegger KA, Binkert CA. Cirse classification system for complications’ reporting: a project evaluation process. Cardiovasc Intervent Radiol. 2024;47(8):1160–2. 38858254 10.1007/s00270-024-03772-3

[9] Hisasue S, Takahashi A, Kato R, Shimizu T, Masumori N, Itoh N, et al. Early and late complications of radical retropubic prostatectomy: experience in a single institution. Jpn J Clin Oncol. 2004;34(5):274–9. 15231863 10.1093/jjco/hyh042

[10] Almogati JG, Ahmed EO. Glycated hemoglobin as a predictor of the length of hospital stay in patients following coronary bypass graft surgery in the Saudi population. Braz J Cardiovasc Surg. 2019;34:28–32. 30810671 10.21470/1678-9741-2018-0202 PMC6385841

[11] O’Sullivan K, Martensson J, Robbins R, Farley KJ, Johnson D, Jones D. Epidemiology of long-stay patients in a university teaching hospital. Intern Med J. 2017;47:513–21. 28145035 10.1111/imj.13379

[12] Ofori-Asenso R, Liew D, Mårtensson J, Jones D. The Frequency of, and factors associated with prolonged hospitalization: a multicenter study in Victoria, Australia. J Clin Med. 2020 22;9(9):3055. 10.3390/jcm9093055 PMC7564707 32971851

[13] Leape LL, Brennan TA, Laird N, et al. The nature of adverse events in hospitalized patients. Results of the Harvard Medical Practice Study II. N Engl J Med. 1991;324(6):377–84. 1824793 10.1056/NEJM199102073240605

[14] Higgins MCSS, Herpy JP. Medical error, adverse events, and complications in interventional radiology: liability or opportunity? Radiology. 2021;298(2):275–83. 10.1148/radiol.2020202341. 33320064 10.1148/radiol.2020202341

[15] Dindo D, Clavien P-A. What is a surgical complication? World J Surg. 2008;32(6):939–41. 18414942 10.1007/s00268-008-9584-y

